# Why and how to embrace AI such as ChatGPT in your academic life

**DOI:** 10.1098/rsos.230658

**Published:** 2023-08-23

**Authors:** Zhicheng Lin

**Affiliations:** Programme of Applied Psychology, School of Humanities and Social Science, The Chinese University of Hong Kong, Shenzhen, Guangdong 518172, People's Republic of China

**Keywords:** artificial intelligence, large language models, ChatGPT/bard, ethics, productivity, open science

## Abstract

Generative artificial intelligence (AI), including large language models (LLMs), is poised to transform scientific research, enabling researchers to elevate their research productivity. This article presents a how-to guide for employing LLMs in academic settings, focusing on their unique strengths, constraints and implications through the lens of philosophy of science and epistemology. Using ChatGPT as a case study, I identify and elaborate on three attributes contributing to its effectiveness—intelligence, versatility and collaboration—accompanied by tips on crafting effective prompts, practical use cases and a living resource online (https://osf.io/8vpwu/). Next, I evaluate the limitations of generative AI and its implications for ethical use, equality and education. Regarding ethical and responsible use, I argue from technical and epistemic standpoints that there is no need to restrict the scope or nature of AI assistance, provided that its use is transparently disclosed. A pressing challenge, however, lies in detecting fake research, which can be mitigated by embracing open science practices, such as transparent peer review and sharing data, code and materials. Addressing equality, I contend that while generative AI may promote equality for some, it may simultaneously exacerbate disparities for others—an issue with potentially significant yet unclear ramifications as it unfolds. Lastly, I consider the implications for education, advocating for active engagement with LLMs and cultivating students' critical thinking and analytical skills. The how-to guide seeks to empower researchers with the knowledge and resources necessary to effectively harness generative AI while navigating the complex ethical dilemmas intrinsic to its application.

## 1. Introduction

Ever-growing scientific advances and data present a significant challenge: a ‘burden’ of knowledge that leaves researchers struggling to keep up with the expanding scientific literature. By contrast, the explosion of knowledge and data is fuelling machine intelligence. The rapid progress in generative AI (see box 1 for a non-technical primer) in the past few years, especially in large language models (LLMs), is a game-changer [1,2]. It is well suited to alleviate the knowledge ‘burden’ and has the potential to revolutionize scientific research. To facilitate the adoption of this new technique and foster discussions and empirical research on the changing landscape of scientific research in the era of generative AI, here I provide a how-to guide for using LLMs in academic settings and offer new perspectives on their implications as informed by epistemology and philosophy of science.

Box 1. **Generative AI, large language models and ChatGPT/Bard**

*Generative AI* trains machine learning (ML) models on a dataset of examples to generate new examples similar to those in the training set, including text, images and music. This generative ability distinguishes it from *predictive AI*, which trains models to predict outcomes on new, unseen data, such as in image classification and speech recognition. Although generative AI dates back to the 1950s, the breakthrough came only recently, thanks to the availability of massive amounts of data and the development of *deep learning* algorithms (‘deep’ refers to the use of multiple layers in artificial neural networks). These algorithms afford the creation of *large language models* (LLMs) to be trained on vast amounts of diverse text data.

Many state-of-the-art LLMs use a type of deep learning algorithm called *transformers* as their backbone. Introduced in 2017, the transformer architecture is a type of deep neural network architecture that uses *self-attention* mechanisms to better process sequential data such as text. Self-attention allows the network to calculate the attention weights between every pair of input elements, effectively allowing the network to weigh the importance of each input element with respect to all other elements. Thus, it allows the network to dynamically focus on different parts of the input sequence and capture long-range dependencies in the data. This mechanism enables it to understand and interpret language in a way that is similar to humans.

One of the most powerful LLMs is Generative Pre-trained Transformer 3 (GPT-3), introduced in 2020 by OpenAI in San Francisco, California. GPT-3 has been trained on a massive amount of text data, allowing it to generate human-like text and excel at challenging natural language processing (NLP) tasks. Recently in November 2022, a derivative of GPT-3 called ChatGPT was launched. It has fine-tuned GPT-3 using *reinforcement learning from human feedback (RLHF)* in a smaller dataset specifically for conversational tasks, making it both conversational and computationally efficient. GPT-3 was updated to GPT-4 and released to the public on 14 March 2023. Another powerful transformer-based LLM is PaLM (Pathways Language Model), developed by Google AI. PaLM has been finetuned to support the chatbot, Bard.

To understand and harness the capacity and potential of generative AI, I will illustrate its capabilities using the popular chatbot ChatGPT. ChatGPT reached 100 million users within just two months of its launch on 30 November 2022. A similar chatbot is Bard, which was launched by Google on 21 March 2023 (see table 1 for a list of other tools). In what follows, I will first identify and elaborate on three features of LLMs, as exemplified by ChatGPT, that make them unprecedentedly apt to augment, if not transform, research life: *intelligent*, *versatile* and *collaborative*. I do so by incorporating specific, practical examples commonly encountered in biomedical and behavioural research. As LLMs are rapidly evolving, I also offer a living resource online, complete with documents that provide tips on crafting effective prompts, examples of usage and relevant links (https://osf.io/8vpwu/).

**Table 1.** A list of AI tools for researchers.

tool	utility	link
ChatGPT (GPT)	multiple-purpose language model	http://chat.openai.com
Wordtune	rewriting text	https://www.wordtune.com
Generate	organizing thoughts, synthesizing information, summarizing text	https://cohere.ai/generate
Codex	completing code	https://openai.com/blog/openai-codex/
Copilot	suggesting code while typing	https://github.com/features/copilot
CoStructure	generating structured tables from unstructured text data, such as scientific articles or contracts	https://costructure.vercel.app
ExplainPaper	providing simple explanations of complex scientific papers	https://www.explainpaper.com
ChatPDF, PandaGPT, Humata	answering questions based on the uploaded pdf file	https://www.chatpdf.com
		https://www.pandagpt.io
		https://www.humata.ai
Elicit	literature search and summary	https://elicit.org

Next, I will critically discuss the limitations of LLMs and, importantly, their ethical and responsible use, as well as implications for equality and education—a debate still in flux. Specifically, I argue that while guidelines for using AI such as ChatGPT in academic research are urgently needed, policing its usage in terms of plagiarism or AI-content detection is likely of limited use. More fundamentally, if AI-created content is deemed valuable based on peer review, there is no reason to reject such content—the identity of the originator of that content is irrelevant from an epistemic point of view. As long as the use of AI is transparently disclosed, there is no need to limit the scope or nature of the assistance it can offer. If, however, the content produced by AI is not original or valuable but still passes peer review, then the problem lies not with AI but with structural issues in the peer review system—AI merely exposes its weaknesses and calls for concerted efforts to improve it. Concerning implications for equality, I contend that generative AI may foster equality for some but exacerbate disparities for others, based on considerations at the individual, group, and national levels. With regard to education, I advocate for the importance of engaging with LLMs and developing critical thinking and analytical skills in students. Given the early nature of generative AI in scientific research, empirical work is scarce, and the views expressed here aim to stimulate further efforts in addressing these important issues.

## 2. Three features of generative AI that make it valuable for researchers

### 2.1. Intelligent

AI is created to perform tasks that typically require human intelligence, including understanding language. According to multiple benchmarks—ranging from Advanced Placement (AP) exams to the Uniform Bar Exam—it is increasingly capable of performing language tasks at a level that matches or surpasses average human performance [3]. Indeed, LLMs such as ChatGPT go beyond generating language to show some form of behaviours that seem to resemble general ‘intelligence’, including problem-solving and reasoning [4].

Formal tests corroborate these observations. For example, in medical question answering, ChatGPT not only achieved accuracy higher than the 60% threshold on the National Board of Medical Examiners (NBME) Free Step 1 dataset—comparable to a third-year medical student—but was able to provide reasoning and informational context [5]. As another example, consider its ability to generate medical-research abstracts based on just the title and journal of the original papers. Not only was there no plagiarism detected, but also human reviewers correctly recognized just 68% of the generated abstracts and wrongly flagged 14% of the original abstracts as generated [6]. These results are remarkable given that they were tested using ChatGPT out of the box. In other words, when the pre-trained model is fine-tuned with a dataset of examples from the relevant domains, the results will be enhanced. Further, as the underlying model (GPT-3.5) is continually being improved (e.g. updated to GPT-4 on 14 March 2023), the performance of ChatGPT is expected to also improve, as demonstrated in medical competency [7].

Whether such performance and behaviour constitute cognitive abilities and can be construed as intelligence of humankind is debated [8]. Indeed, human intelligence is a latent construct that does not yield itself to a straightforward measure in non-human animals and machines, not least because traditional intelligence tests such as Intelligence Quotient (IQ) are anthropocentric—designed specifically for humans. Even within human populations, IQ tests need to be significantly altered for testing in children and people with disabilities. Thus, to better understand the nature of AI and measure its progress in obtaining intelligence, much research is needed to define intelligence and measure it in a way that is comparable and fair across machines and mankind [9].

Given the controversy, the term intelligence will be used here to refer to artificial intelligence, regardless of whether that might be considered true human intelligence or not. Indeed, for practical purposes—that is, from an end user's perspective—such debates are mostly moot so long as AI is able to get the job done. To appreciate the intelligence of AI, perhaps the most straightforward way is to have a conversation with ChatGPT (for a practical guide to its efficient use, see box 2). ChatGPT is strikingly human-like: it ‘understands’ text input and responds to it like a well-learned person—and in some ways, perhaps better than most people. The implications are likely to be profound, as the cost of intelligence has never been so low. This makes LLMs such as ChatGPT incredibly empowering for organizations and individuals.

Box 2. **A practical guide to the efficient use of ChatGPT/Bard**

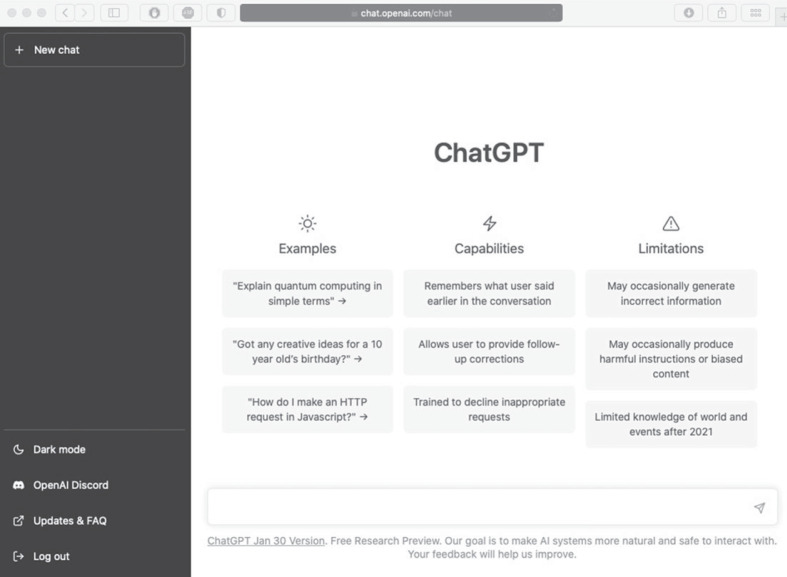

ChatGPT can be accessed through a web interface. To get started, go to the official webpage (https://chat.openai.com) and sign up for an OpenAI account (phone verification is required). Once logged in, you will see its interface, as shown above, where you will find example prompts to ask the chatbot and its capabilities and limitations. Interact with the chatbot by typing your prompt in the blank input bar (bottom) or initiating a new chat (top left).

To use it more efficiently, familiarize yourself with three key features. First, each prompt in your chat history has an *edit* button when you hover over it (on the right), where you can edit your previous prompt. After your edit, the chatbot will provide a new response accordingly. This is useful when your initial attempt does not yield the response you want. Second, you can provide *feedback* on the response (thumb up and thumb down icons, on the right) and you can ask it to *regenerate* responses (bottom)—which you can toggle to compare and find the most desirable one. Third, you may want to start a *new chat* for each project, as ChatGPT takes into consideration the chat history of each conversation.

Getting the desired results may require some thought. That is, feed it the right prompts (see six tips for writing effective prompts in the online supplemental materials: https://osf.io/8vpwu/). LLMs tend to make assumptions about user intent based on the prompt given, rather than asking clarification questions. To enhance accuracy, it is important to provide it with sufficient contextual information [10]. In general, prompts should be clear and concise. You can provide very *specific* instructions and offer feedback and new directions as follow-ups throughout the conversation. For example, you may ask it to explain a statistical concept by typing: ‘Explain Cook's distance’. Suppose you find the response a bit dense. You can follow up by typing: ‘Can you explain it like I am five?’ As another example, you can feed it with your writing and ask it to make it more concise: ‘Please rewrite it to be more concise’. But if you find the rewrite a bit non-sophisticated, you can follow up with a prompt like: ‘Please make it more sophisticated for an educated audience’. You can keep fine-tuning it to your desire. However, if you have a clear goal, using an elaborate, specific prompt will work best. In fact, you can enlist ChatGPT to help improve the prompt (e.g. ‘Please evaluate each prompt I present and provide a rating on a scale of 1 to 5, based on its clarity and level of engagement. Kindly provide constructive feedback on how I can improve each prompt if necessary. Should the rating for a prompt be 4 or above, proceed to answer it; otherwise, create a new prompt that meets the desired criteria’).

ChatGPT is helpful for many things, from helping you learn, code, analyse and write to assisting with your teaching, mental needs and job applications. Ultimately, to get the most out of its capabilities, be creative and imaginative. Say you have written an emotional email. Before you send it, you can enlist ChatGPT to check its tone, using the following prompt: ‘Acting as an editor, please make recommendations on how to improve the email below using the principles and concepts of Nonviolent Communication (NVC). For each edit, please provide the rationale and some examples’. Indeed, you can ask ChatGPT to act as a simulated patient, therapist, coach, advisor, tutor, professor or interviewer—the possibilities are endless. Or consider your next job application. You can request ChatGPT to help craft a customized cover letter for the job, using a prompt like: ‘Please write a cover letter for the job description below using my CV that follows’.

Example screenshots of using R and Adobe Illustrator, tips for writing effective prompts, and a living resource are provided online (https://osf.io/8vpwu/). This guide also applies to the chatbot, Bard, which is highly similar to ChatGPT except for some minor differences (e.g. the ‘[r]egenerate response’ function in ChatGPT is replaced by the ‘[v]iew other drafts’ function in Bard).

For knowledge workers, it enables us to be more productive and efficient—doing more with less. A list of tips, examples and resources is provided online (https://osf.io/8vpwu/). For example, ChatGPT can provide explanations and help us learn a new domain more efficiently (e.g. ‘Act as an R instructor and teach me the basics'), write and debug codes faster (e.g. ‘Write R code to do a one-way ANOVA based on the following data’), assist with writing (e.g. ‘Rewrite the following paragraph to be more concise’) and more. By automating aspects of the research process and improving research efficiency, ChatGPT helps to accelerate the pace of scientific discovery.

From the perspective of philosophy of science, AI also has the potential to uniquely complement and enhance human intelligence in facilitating scientific inquiry and discovery. For one, by analysing and synthesizing vast amounts of data from different fields, LLMs may help to discover connections between seemingly disparate fields—connections that might not be immediately apparent to human researchers. For another, whereas human researchers are inevitably influenced by personal values and preferences, social norms and cultures, and historical assumptions and biases [11], LLMs do not have emotions, consciousness or personal motivations. Indeed, by analysing vast and diverse amounts of data with the same algorithmic process, LLMs have broader perspectives and greater consistency than individual researchers, thus reducing the risk of cognitive bias, from confirmation bias to the availability heuristic. Moreover, although biases do exist in LLMs due to the training data and algorithms—a limitation discussed later—these biases are not identical to human biases and can help to counteract or reduce certain predispositions in scientific practices, potentially improving the reliability and objectivity of scientific inquiry [‘strong objectivity’; 12].

### 2.2. Versatile

As alluded to before, what makes generative AI such as ChatGPT special is that it excels not just in one domain but across many domains, thanks to the diverse training text data. ChatGPT has been trained to understand and generate cohesive text across a broad spectrum of subjects, from general knowledge to specific areas such as science and mathematics. It is proficient in a wide range of human languages (English, Spanish, French, German, Italian, etc.) and computer programming languages (Python, JavaScript, Java, C++, R, etc.). This versatility makes it useful in multiple capacities, such as a coach, research assistant and co-writer.

Consider the many tasks that researchers perform every day. In administrative roles, writing and editing documents and emails can benefit from ChatGPT. In teaching, generating questions and grading them, creating discussion points and questions, editing syllabuses and handouts—these are some common tasks that can also use help from ChatGPT. In research, too, practically all processes—other than those involving physical interactions—can enlist ChatGPT. Indeed, formal evaluations in finance research show that ChatGPT can significantly assist with idea generation, data identification and more. Incorporating private data and domain expertise can further improve the quality of the output [13].

For example, ChatGPT can help with familiarizing oneself with new topics (e.g. ‘What is generative AI’), summarizing (e.g. ‘Summarize the key issues mentioned below in a table, using two columns: ‘Ethical issue’ and ‘Key question’’), coding (e.g. ‘The following code has errors. Can you advise how to fix it’), brainstorming (e.g. ‘Write five titles based on the following keywords’), providing feedback (e.g. ‘Act as a journal reviewer and provide feedback on the abstract below’) and more.

### 2.3. Collaborative

ChatGPT is also special for its conversational capability, thanks to a method called reinforcement learning from human feedback (box 1). This capability makes it an excellent collaborator, able to listen and update its responses based on user feedback. To illustrate, suppose we want to improve our writing. We can start with the prompt: ‘Act as a copy editor, revise the text below and explain your edits’. If we don't like a particular expression in the revision, we can follow up with a new request: ‘Can you make ‘…’ more elegant?’ Indeed, we can ask ChatGPT to give the writing some personality, revise it for an academic audience, make it more persuasive or assertive, in the style of Hemingway, and so on. From proofreading to editing and rewriting, the possibilities are endless.

The utility of intelligent, versatile, always-on collaboration afforded by ChatGPT cannot be overstated. It offers a great channel to bounce ideas off of. It also helps to alleviate common drudgery and mental block—making research more fun. For example, regular expressions (regex or regexp) are a powerful tool commonly used in text analysis to define patterns for strings—thus enabling matching, extracting, and substituting patterns—but they can be complicated and error-prone. ChatGPT makes it much easier to use regex by helping researchers understand the syntax and usage (e.g. ‘How to replace all occurrences of Ph.D. with PhD in R using regex?’), and then construct or refine a regex (e.g. ‘Test the regex on a sample text and return the matched substrings’). Similarly, consider a common mental block: writer's block. ChatGPT helps by brainstorming and collaborating with us, starting the first step that ultimately paves the way for a thousand-mile journey to publication (e.g. ‘Give me five ideas to begin an article on ‘how AI may help researchers’’).

## 3. Limitations of generative AI

As with any other tool, generative AI has limitations. These limitations are rooted in the principles and techniques that make it so powerful in the first place (box 1). Specifically, LLMs such as ChatGPT are language models trained on massive data. When they respond to queries and engage in conversation, they do not understand the content in the same way humans do, but rather make predictions about text based on patterns learned from training. They ostensibly write like an educated human—a great achievement—but they are not. This will become plainly clear once we interact with them in a deep manner (e.g. they can contradict themselves at times, and they do not have a strong grasp of context). The important point, however, is to use them as powerful tools rather than relying on them.

In the context of research aid—such as for a research project or for lecturing on a topic—a major limitation of LLMs is that they may fabricate facts, creating confident-sounding statements and legitimate-looking citations that are false (hallucination). Thus, as with any other source of information (e.g. Wikipedia), it is important to critically evaluate and verify AI responses, particularly when reliability is critical [14]. An important next step might lie in developing methods to quantify and signal the epistemic uncertainty and potential limitations of AI-generated results.

Still another limitation has to do with the training data for LLMs. These data are not—and cannot be—truly neutral or objective, but rather laden with assumptions and biases, ranging from political and ideological to cultural [12,15]. From the perspective of standpoint epistemology, such biases and assumptions are not inherently problematic. To the extent that knowledge is socially situated—different people have different experiences and perspectives that shape their understanding of the world—biases and assumptions can be understood as reflective of specific *standpoints* (i.e. perspectives) of the people who generated and compiled the data.

Yet, the challenge is that the standpoints represented in the training data may not be evenly distributed or representative of all perspectives. Indeed, the issue of underrepresentation in knowledge production has been widely documented, including the underrepresentation of certain racial, ethnic, gender, political and geographical groups as participants and researchers in medical and scientific research [16,17]. Lack of diversity in the research process contributes to prejudices, stifles epistemological plurality, and limits the range of topics and questions being pursued [11]. In turn, biases and limitations in the data may be picked up—or even amplified—in LLMs. For example, when the training data predominantly reflect the views and experiences of certain groups (e.g. people from Western, educated, industrialized, rich and democratic societies), then the LLMs trained on these data will inevitably reflect these biases. This uneven representation can lead to a reinforcement of dominant perspectives and marginalization of others, creating a potential for bias in the outputs of these models.

There are additional limitations in using AI/LLMs to aid teaching and administrative tasks. In the realm of teaching, one potential use of AI is grading [18]. While such an application might seem promising in terms of efficiency, establishing a system that grades objectively, reliably and fairly presents significant challenges. To ensure fairness and accuracy, the AI’s grading algorithms would need to be based on clear, comprehensive rubrics—a non-trivial task in itself. Even then, potential biases in the AI’s interpretation of student work could lead to discrepancies in grading. Furthermore, nuances of student creativity and originality, which are often the hallmarks of exceptional work, might be overlooked or misinterpreted by an AI grader. Therefore, human supervision and verification are necessary safeguards in the grading process, potentially reducing the time and labour-saving benefits of the AI.

In the administration domain, AI is useful for drafting emails and similar tasks. While AI can be used to streamline the process and improve efficiency, it can also backfire in sensitive situations, when human touch is what matters most—something that cannot be replaced by AI. One case that underscores this limitation is a recent incident at Vanderbilt University, where two deans used ChatGPT to draft an email to students about a mass shooting at Michigan State University. Their use of AI in this sensitive situation led to their suspension, illustrating the potential pitfalls of over-reliance on AI for sensitive administrative tasks. Thus, striking a balance between leveraging AI's efficiency and maintaining the human touch that is often essential in academic settings will be an ongoing challenge in the implementation of these technologies.

## 4. Implications of generative AI: ethical use, equality and education

### 4.1. Ethical and responsible use

The power of generative AI such as ChatGPT raises many thorny questions regarding its ethical use, from plagiarism, image manipulation, authorship and copyright to fake research (table 2). It is one thing to ask it to act as an editor to correct language issues in our own writing, but quite another to ask it to write an entire paragraph and then copy it [2]. The former is similar to the services offered by other writing tools and university writing centres, while the latter is widely regarded as plain plagiarism. However, the boundary between acceptable help and too much help is not always clear-cut. When we feed ChatGPT with our own text and ask it to rewrite it, is that too much help to be considered ethical? Does the answer depend on the length of the text—and if so, how can we determine the proper boundary? The same questions apply to text-to-image AI (e.g. DALL·E 2, Midjourney, Stable Diffusion). Is it okay to use AI-generated images in the paper, or would that be considered plagiarism? And in the cases where AI offers ‘too much’ help, can it be listed as a co-author? Fundamentally, who has the right to claim copyright over AI-generated content (text, images, etc.): the prompt creator, the AI, the AI developer or the owners of the training data?

**Table 2.** An agenda for the ethical and responsible use of AI in scientific research.

ethical issue	key question
plagiarism	How much help from AI is too much help?
AI authorship	Can AI be listed as a co-author? If not, how to properly document and acknowledge its contributions?
copyright of AI-generated content	Does the AI-generated content belong to the prompt creator, the AI tool, the tool creator, or the owners of the training data?
fake research and fraudulent papers	How to detect AI-generated content effectively?

These questions are important for the community to consider and address. Currently, publishers and journals are divided in their policy and stance on some of the questions. For example, Springer Nature does not allow LLM tools to be listed as authors, and requires researchers to document their use in the paper [19]. On the other hand, *Science* family journals not only ban AI tools as authors, but also prohibit the use of AI-produced content (text, images, figures, graphics) in the paper [20]. Although such swift decisions are understandable, going forward it is important to engage the whole scientific community to reach a more consistent and informed consensus. For example, banning AI tools as authors because of their inability to take responsibility flies in the face of the long-standing practice of posthumous authorship [1].

The more practical issue is that it may not even be feasible to detect AI-generated content with sufficient accuracy to be useful. Compared with typical AI-generated content, human-generated content generally—but not always—has higher *burstiness*, mixing longer or more complex sentences with shorter ones, and with higher *perplexity*, using words that are less expected [21]. However, some human writers do write with low burstiness and perplexity, posing a problem of false positives for algorithms. Moreover, LLMs can be instructed to write content with higher burstiness and perplexity, creating a problem of false negatives for algorithms. On top of that, given that LLMs are constantly evolving and improving, it is reasonable to assume that their ability to evade detection may do so as well. Thus, although algorithms for detecting AI content may be useful to compare different groups of writing, they are unlikely to be able to ‘convict' any individual writing. Banning the use of AI-generated content may prove challenging to implement.

Fundamentally, if AI-created content is valuable, there is no reason to reject such content. From an epistemic point of view, we should not treat a finding differently just based on the status of the author, whether it is a Nobel-prize winner or a junior academic member. The identity of the author is irrelevant. The same applies to AI: if AI has valuable, original content, there seems no epistemic reason to devaluate it just because it is created by AI. The real question is the vetting of its value—which rests on the human author and reviewers. Thus, a more pragmatic approach to AI in academic publishing is to encourage or mandate its transparent use [22] rather than banning it outright or even limiting it. From this perspective, there is no need to limit the amount or kind of help from AI—no concept of too much help from AI—as long as it is transparently reported.

Perhaps a more urgent issue with AI concerns its potentially serious threat to scientific integrity: the inevitable exponential rise of AI-generated, fraudulent papers submitted to scientific journals—some of which will pass peer review and become part of the scientific literature. Paper mills, which are already notorious for creating and selling fake research with fraudulent data and images, will become an even bigger threat when equipped with the unprecedented power of AI [10]. However, the negative disruptions brought about by AI, as with the advent of any other powerful tool in history, are to be expected. Indeed, more generally, if content that is not valuable or simply fake can pass peer review, whether it is from AI or not, the problem has more to do with the peer review system. The potential negative impact is not a cause to forbid or limit the use of AI, but a call to step up our efforts in implementing better practices in scientific review and publishing.

Such practices may involve the implementation of rigorous and open peer review (e.g. published peer review exchanges), collaborative review (e.g. discussions among reviewers and the action editor before making an editorial decision) and open science practices (e.g. open data and materials). These practices serve to deter fraudulent submissions, as through open review, the review process is subject to scrutiny by the wider scientific community; they also enhance the probability of detecting fraudulent content, as the accessibility of data and materials simplifies the process for others to validate the results. For these practices to be most effective, researchers need to be aware of the potential for AI tools to be used to generate fraudulent content, as well as to be alert to potential signs of such fraudulent content. Thus, education and awareness are vital. In addition, AI-based tools may be developed to detect patterns indicative of data fabrication or falsification, as well as to identify inconsistencies or errors in data analysis. Together, these strategies can help mitigate the negative impact of AI on knowledge production and improve the accuracy of the scientific record more generally.

### 4.2. Impacts on equity

Having discussed the strengths, limitations and ethical use of generative AI, a natural question arises concerning its implications for equity. Perhaps paradoxically, the availability of powerful, versatile AI tools can promote equality for some while amplifying disparities for others. On the one hand, a main contributor to global disparities in scientific research is language; for example, most mainstream journals are in English, bestowing a natural advantage on native English researchers [16,17]. LLMs can help level the linguistic playing field by offering a language boost for non-native English researchers through copy editing and other writing assistance (e.g. ‘Act as a copy editor, proofread the following text for an academic journal, and highlight the changes at the end’). Thus, researchers previously disadvantaged in the English language can now compete on a more equal footing.

On the other hand, there are reasons to believe that LLMs may also exacerbate existing disparities. To the extent that LLMs can boost research productivity, such a boost may favour researchers who are already advantaged, as exemplified at the individual, group and national levels. At the individual level, researchers who are already skilled at tasks for LLMs are likely to reap more benefits. This is because LLMs are not magic machines that can automatically crank out papers or code for us, but rather valuable tools that require learning and understanding on the user's part, just like any other tool. Consider coding. Although LLMs can aid beginners in learning how to code and provide solutions to some problems, ultimately researchers need to know how to ask LLMs to perform the task and then comprehend the output—skills that require understanding and mastery. Thus, to the extent that coding skills give researchers a leg up, this advantage is amplified with the help of LLMs, enlarging the divide between coders and non-coders.

At the group level, researchers with the resources to assemble a large team are poised to benefit more from the productivity boost, as team members become more efficient and productive with the help of LLMs. Consider a team of two and a team of 10—a difference of 8. Suppose the productivity of each person is multiplied by 1.5 with LLMs: then the difference becomes 12. In other words, existing disparities are multiplied by LLMs. At the national level, access to LLMs is not even but prioritized toward leading Western industrialized nations; for example, as of 20 July 2023, ChatGPT and Bard are not available in regions such as mainland China and Hong Kong. Indeed, when Bard was launched in March 2023, it was only available to users in the USA and UK. Even nations that can access LLMs may not benefit as much from them due to a host of factors, such as Internet access and the varying capabilities of LLMs in different languages. Thus, the unequal multiplication of productivity afforded by LLMs may amplify existing disparities between nations.

### 4.3. Education

The inherent limitations and ethical concerns of LLMs raise questions about how to engage with them in education [18]. Given their power and utility, it is crucial that educational strategies focus on preparing students to harness the capabilities of LLMs without succumbing to their limitations, rather than resorting to outright bans on their use. One key objective in this regard is to help students develop critical thinking and analytical skills, enabling them to evaluate outputs generated by LLMs with special attention to their accuracy, reliability and potential biases. This is only possible when LLMs are integrated into curricula as an integral part of education.

Indeed, according to constructivist learning theory, learning is an active, constructive process where learners build their own understanding by connecting new information to their existing knowledge [23]. LLMs can serve as powerful tools, providing students with vast amounts of information and diverse perspectives. This allows students to engage in active learning by interacting with LLM outputs, relating them to what they already know, evaluating the outputs and revising their understanding accordingly. On the other hand, learning also benefits from guidance and social interaction, as emphasized in Vygotsky's zone of proximal development (ZPD) theory [24]. Learning, according to this theory, occurs most effectively in the ‘zone’ between what a learner can do independently and what they can do with help. By using LLMs as learning tools, educators can guide students through complex concepts and tasks, gradually withdrawing their support as students develop the skills to evaluate LLM outputs independently. Integrating LLMs into curricula allows educators to serve as ‘more knowledgeable others’, providing assistance and resources to extend students' learning beyond what they could achieve alone in using LLMs.

Such integration can take multiple forms, including hands-on training with LLMs (e.g. interacting with LLMs in diverse learning activities), case studies (e.g. dissecting real-world examples to illustrate potential benefits and limitations of LLMs), teaching AI ethics and literacy (e.g. bias, transparency, privacy and societal impacts), evaluating LLM-generated content (e.g. assessing its quality and reliability) and fostering collaboration with LLMs (e.g. examining how human intelligence and LLMs may work together to create better results across different topics and fields of study). Doing so can help encourage students to think critically about the role of LLMs in their work and acquire skills for effective collaboration with them. Building these skills benefits from a strong foundation in scientific reasoning, research methodology and subject-specific knowledge—all within the realm of traditional education.

Case studies are emerging to help illustrate the potential of LLMs in facilitating teaching and learning in language, computer science and medicine. For example, ChatGPT can be used to promote engaging and adaptive language teaching and learning [25], to assist teaching and learning in programming courses in computer science curriculum [26] and to support medical education during the preclinical and clinical years [27]. These cases highlight the potential of AI in facilitating teaching and learning as well as the importance of acquiring digital competence in reaping the benefits of AI. Thus, integrating LLMs into curricula is not an option but a must, if we are to foster digital competence in students and faculty.

## 5. Summary and concluding remarks

Irrespective of our attitudes and ethical implications, generative AI such as LLMs is here to stay. Like other powerful tools invented in history, such as the Internet and personal computers, generative AI is posed to have measurable short-term effects and potentially transformative long-term effects. As knowledge workers, it is in our best interest to embrace LLMs like ChatGPT to augment our skills, creativity and productivity [14]. In this how-to guide, I have identified and elaborated on three characteristics that make LLMs valuable: intelligent, versatile and collaborative. Since learning to write effective prompts to interact with LLMs is likely to become an indispensable skill, I have also offered practical tips, examples and resources to get started (e.g. box 2 and online materials at https://osf.io/8vpwu/).

At the same time, to ensure the ethical and responsible use of generative AI in research, I argue that transparent reporting is crucial (table 2); however, from technical, philosophical and epistemic standpoints, there is no need to limit the type or amount of assistance that LLMs can provide. Although this concept might seem outlandish, it is relatively common in art. For example, Andy Warhol maintained authorship for many of his paintings that were created by other artists and machines, by providing the ideas for the artwork. Similarly, some writers retain authorship for books in which they provide the story concepts and characters, with the prose completed by other writers.

However, I have also identified three major challenges posed by LLMs. The first concerns the evaluation of output from LLMs, which should be explicitly dealt with in the context of education, by developing critical thinking and analytical skills in students. The second has to do with the potential proliferation of fake research, which may be addressed through open science practices (e.g. open peer review, data, code and materials) in conjunction with education and the development of AI-based tools. The third challenge stems from the potential exacerbation of disparities, which may not have a straightforward solution. Continuously grappling with this issue will be crucial in determining how it unfolds.

As generative AI continues to advance, it will challenge our understanding of and practices in knowledge production and dissemination. On the one hand, it urgently underscores the importance of diversity and inclusivity in the training data, which can help to enhance the reliability and objectivity of the insights generated by LLMs, moving us closer to the goal of ‘strong objectivity’ as proposed by Sandra Harding. On the other hand, how we embrace and manage the transformative potential of generative AI will shape the future of scientific research and education. It is incumbent upon us to effectively integrate LLMs in research and education, to engage with the complex ethical and practical issues brought forth by these evolving technologies. This how-to-guide contributes to the ongoing conversation by providing practical resources and new perspectives from epistemology and philosophy of science.

## Data Availability

No data are used.
